# Single Cell Dissection of Epithelial-Immune Cellular Interplay in Acute Kidney Injury Microenvironment

**DOI:** 10.3389/fimmu.2022.857025

**Published:** 2022-05-04

**Authors:** Min Zhang, Lingling Wu, Yiyao Deng, Fei Peng, Tiantian Wang, Yinghua Zhao, Pu Chen, Jiaona Liu, Guangyan Cai, Liqiang Wang, Jie Wu, Xiangmei Chen

**Affiliations:** ^1^ Department of Nephrology, First Medical Center of Chinese People’s Liberation Army (PLA) of China General Hospital, Nephrology Institute of the Chinese People’s Liberation Army, State Key Laboratory of Kidney Diseases, National Clinical Research Center for Kidney Diseases, Beijing Key Laboratory of Kidney Disease Research, Beijing, China; ^2^ Department of Nephrology, Guizhou Provincial People’s Hospital, Guiyang, China; ^3^ Department of Ophthalmology, Ophthalmology & Visual Science Key Lab of People’s Liberation Army (PLA) of China, General Hospital of Chinese People’s Liberation Army (PLA) of China, Beijing, China

**Keywords:** acute kidney injury, renal tubular epithelial cells, microenvironment, intercellular crosstalk, trajectory analysis

## Abstract

**Background:**

Understanding the acute kidney injury (AKI) microenvironment changes and the complex cellular interaction is essential to elucidate the mechanisms and develop new targeted therapies for AKI.

**Methods:**

We employed unbiased single-cell RNA sequencing to systematically resolve the cellular atlas of kidney tissue samples from mice at 1, 2 and 3 days after ischemia-reperfusion AKI and healthy control. The single-cell transcriptome findings were validated using multiplex immunostaining, western blotting, and functional experiments.

**Results:**

We constructed a systematic single-cell transcriptome atlas covering different AKI timepoints with immune cell infiltration increasing with AKI progression. Three new proximal tubule cells (PTCs) subtypes (PTC-S1-new/PTC-S2-new/PTC-S3-new) were identified, with upregulation of injury and repair-regulated signatures such as Sox9, Vcam1, Egr1, and Klf6 while with downregulation of metabolism. PTC-S1-new exhibited pro-inflammatory and pro-fibrotic signature compared to normal PTC, and trajectory analysis revealed that proliferating PTCs were the precursor cell of PTC-S1-new, and part of PTC-S1-new cells may turn into PTC-injured and then become fibrotic. Cellular interaction analysis revealed that PTC-S1-new and PTC-injured interacted closely with infiltrating immune cells through CXCL and TNF signaling pathways. Immunostaining validated that injured PTCs expressed a high level of TNFRSF1A and Kim-1, and functional experiments revealed that the exogenous addition of TNF-α promoted kidney inflammation, dramatic injury, and specific depletion of TNFRSF1A would abrogate the injury.

**Conclusions:**

The single-cell profiling of AKI microenvironment provides new insight for the deep understanding of molecular changes of AKI, and elucidates the mechanisms and developing new targeted therapies for AKI.

## Introduction

Acute kidney injury (1–3) (AKI) is a serious health risk, characterized by an abrupt loss of renal function, which is also a leading cause of chronic kidney disease (CKD) and end-stage renal failure. AKI commonly caused by ischemia, sepsis, or nephrotoxic insult, which result from a variety of conditions, including major surgery, sepsis, trauma, dehydration, and toxic drug damage. The incidence of AKI has gradually increased in recent years, with a relatively high incidence of 3%-5% among the general population in hospitals which can be as high as 30%-60% in the intensive care unit (ICU). The mortality rate can be as high as 60-70% when combined with multi-organ failure which has caused a great economic and mental burden for patients and society (4).

AKI is usually characterized by pathological alterations (5–7) such as proximal tubule cells (PTCs) dysfunction or death, triggering a poorly understood autologous cellular repair program. Also, infiltrating immune cells (8–13) undergo phenotypic and functional changes in response to AKI injury and repair processes. Studies (14–17) using bulk transcriptional profiling have characterized molecular characteristics associated with kidney injury and recovery. The transcriptional average across cell populations was revealed, which may hide or skew signals of interest with specific cellular identities and biologically-relevant mechanisms. Dissecting the molecular basis associated with AKI microenvironment changes and the complex cellular interaction is essential to elucidate the mechanisms and develop new targeted therapies for AKI.

Single-cell RNA-sequencing (scRNA-seq) (18–24) is a powerful technology capable of revealing the heterogeneous cellular and molecular characteristics along with the disease initiation and progression. Recent studies for AKI, using scRNA-seq, identified novel cell-subtypes with diverse transcription phenotype response to AKI (9, 12, 24). Additionally, infiltrating immune cells such as Treg (12) and myeloid cells (9) associated with AKI injury and repair were identified. What remains unknown is how infiltrating immune cells influence the process of AKI damage and repair.

Here, we report findings from scRNA-seq analysis of 52,162 cells from mouse kidney tissue samples at 1, 2 and 3 days after ischemia-reperfused AKI and healthy control. We uncovered three PTC-new subtypes and found that PTC-S1-new exhibited pro-inflammatory and pro-fibrotic signature. Proliferating PTCs were the precursor cells of PTC-S1-new, and part of PTC-S1-new cells may turn into PTC-injured and then become fibrotic in the case of sustained damage. Also, the PTC-injured highly expressed Tnfrsf1a and Kim-1 and interacted closely with macrophage and monocyte through Tnfrsf1a/Tnf signaling axis. Functional experiments revealed that the exogenous addition of TNF-α dramatically promoted kidney inflammation and injury and specific depletion of TNFRSF1A would abrogate the injury.

## Results

### Dynamic Changes of Cellular Proportion and Phenotype During the IRI AKI

**Figure 1:**
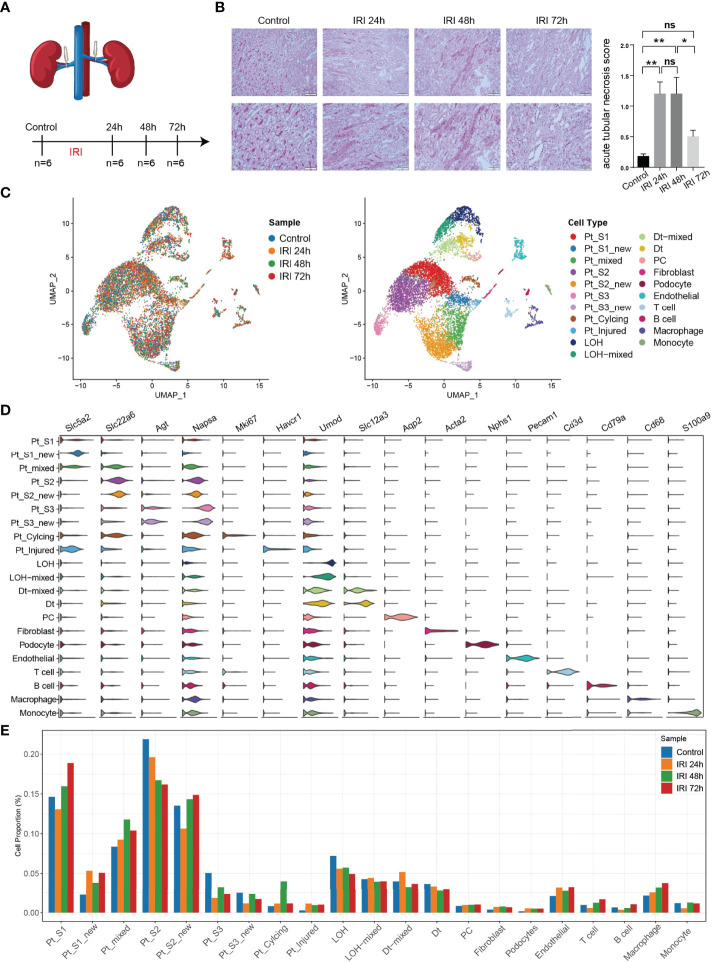
An overview of single cell transcriptomic atlas of mouse IRI kidney. **(A)** Summary of experimental strategy. n = 6 mice per group. **(B)** The histopathology images and acute tubular necrosis score in control, IRI 24h, IRI 48h and 72h, One-way ANOVA followed by Turkey’s multiple comparisons was used for three or more groups comparisons. *p<0.05, **p<0.01. **(C)** The distribution of 52,162 high-quality kidney cells in distinct sample timepoints (left) and cell types (right). **(D)** Cell type annotation and representative marker genes expression in 21 cell types. **(E)** Relative contribution of each cell type in distinct sample timepoints. ns, Not Statistically Significant.

To obtain a comprehensive cellular atlas alongside AKI progression, the kidney sample of mice at 1, 2 and 3 days after ischemia-reperfused AKI and healthy control were subjected for scRNA-seq and each group contained 6 samples (**Figure 1A**). The renal tubules show obvious damage at 24 hours after IRI, mainly manifested by vacuolar degeneration of renal tubular epithelial cells, partial detachment of brush border and disordered cell arrangement. At 48 hours, histopathology images show severe detachment of renal tubular epithelial cells, the appearance of cellular tubular pattern and protein tubular pattern, and at 72 hours, some renal tubular epithelial cells appear to regenerate and rearrange. Compared with the control group, the acute tubular necrosis score and serum concentrations of serum creatine (Scr) and blood urea nitrogen (BUN) increase significantly at 24 hours and 48 hours after IRI, and decrease with the initiation of tubular epithelial cell regeneration and repair at 72 hours after IRI (**Figure 1B** and **Supplementary Figure 1**). In parallel with the acute tubular necrosis score, the serum concentrations of Scr and BUN recover to baseline level at 72 hours after IRI (**Figure 1B** and **Supplementary Figure 1**). The single-cell suspensions of six mice were pooled together in each single cell experiment and then loaded onto one microfluidic chip to generate the complementary deoxyribonucleic acid (cDNA) library following the method in Conway BR., et al (9). A total of 52,162 high quality cells passed quality control (methods, **Supplementary Figure 2**) and these cells could be defined as 21 cell subtypes including epithelial cells such as PTCs, distal tubular cells, podocytes, principal cells, endothelial cells, fibroblast, and immune cells such as T cells, myeloid cells (**Figure 1C**). Most sequenced cells were PTCs, three new PTC subtypes (PTC-S1-new/PTC-S2-new/PTC-S3-new), and mixed cell types (expressing markers of different renal cell types) are identified (**Figure 1D**). We found that the portion of normal PTCs (PTC-S1, PTC-S2, PTC-S3) change dramatically along with AKI progression (**Figure 1E**), indicating that AKI results in PTCs dysfunction or death, and also AKI triggered the PTC to repair itself on the other, therefore, novel PTC subtypes would generate. Additionally, the portion of immune cells expanded in the kidney along with AKI (**Figure 1E**) indicate the important role of immune cells in AKI (9).

### Pro-Inflammatory and Pro-Fibrotic PTCs Subtypes Expanded in AKI

**Figure 2:**
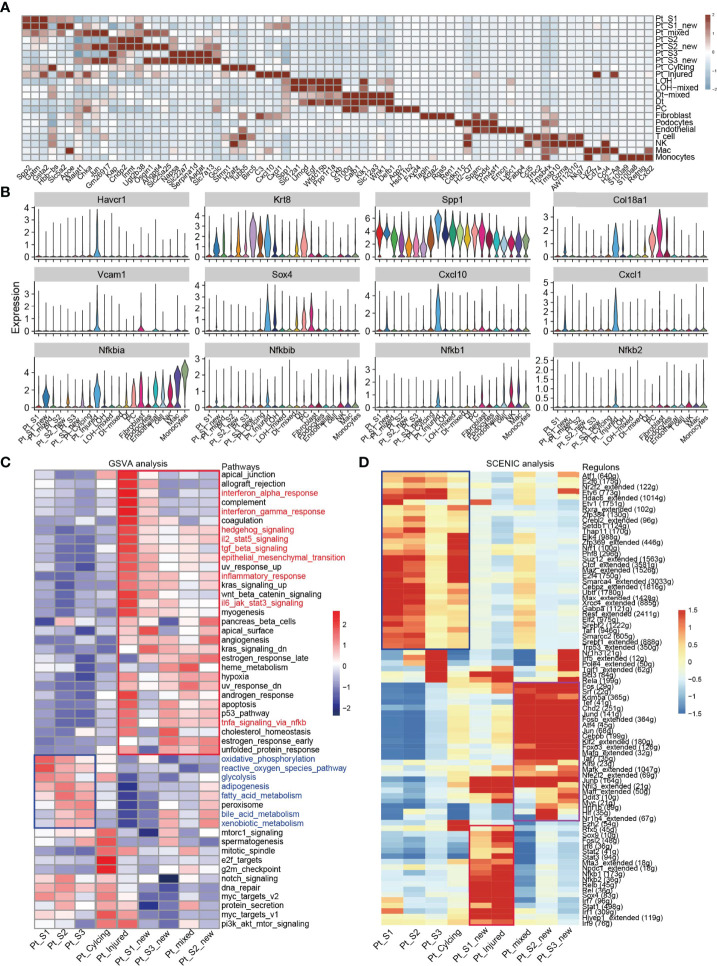
Transcriptomic features of distinct cell types. **(A)** Top 4 differentially expressed genes in 21 cell types. **(B)** The expression level of representative kidney injury and inflammatory signatures in 21 cell types. **(C)** Enriched signaling pathways in PTC-new, injured PTC and normal PTC, estimated by GSVA. **(D)** Enriched regulons in PTC-new, injured PTC and normal PTC, estimated by SCENIC.

**Figure 3:**
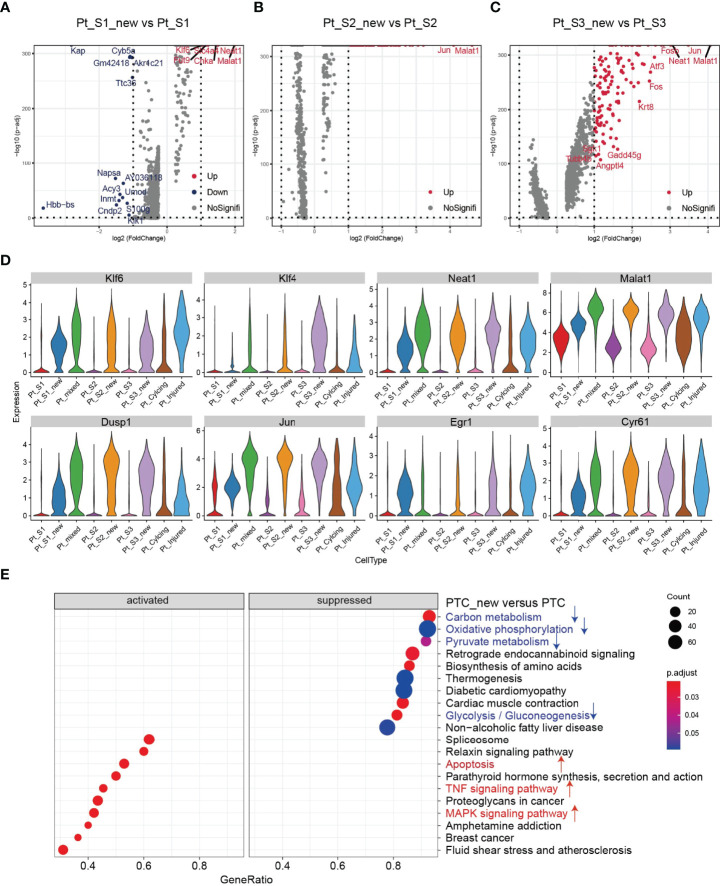
Transcriptomic features of PTC-new subtypes. **(A–C)** Volcano plot showing the differentially expressed genes in PTC-S1-new versus PTC-S1 **(A)**, PTC-S2-new versus PTC-S2 **(B)**, PTC-S3-new versus PTC-S3 **(C)**. **(D)** The expression level of representative kidney injury or repair regulated signatures in PTC subtypes. **(E)** Enriched gene ontogeny (GO) among PTC-new subtypes versus normal PTC.

After annotation of cell subtypes, we then compared the molecular characteristics and the transcription correlation of PTC subtypes. As reported previously, the PTC-injured cells exhibited a high expression level of injury associated signature such as Havcr1 (Kim-1), Krt8, Spp1, Spp2, Vcam1(**Figures 2A, B**). In this study, we also found that the PTC-injured and part of PTC-new cells expressed a high level of pro-inflammatory and pro-fibrotic signatures such as Cxcl10, Cxlc1, Nfkbia, Nfkbib, Nfkb1, Nfkb2, and Col18a1 (**Figure 2B**) Also, the portion of PTC-injured and PTC-S1-new cells elevated in the kidney along with AKI, indicated that pro-inflammatory and pro-fibrotic PTCs subtypes expanded in AKI. Additionally, the injured PTCs had a higher expression level of antigen-presentation genes such as H2-Aa and H2-Eb1 (**Figure 2A** and **Supplementary Figure 3**), indicating that PTCs would exhibit part time immunization features under injured condition. We also compared the functional difference and enriched signaling pathways among PTC subtypes and we found that pro-inflammatory signaling pathways such as Interferon alpha and gamma, IL2 stat5 signaling, TGF beta signaling, IL6 jak stat3, and TNF were mainly enriched in PTC-new and PTC-injured, with highest expression level in PTC-injured (**Figure 2C**). PTC-new and PTC-injured showed impairment of metabolism signaling pathways such as oxidative phosphorylation, glycolysis, fatty acid metabolism, and xenobiotic metabolism (**Figure 2C** and **Supplementary Figure 4**). Additionally, the single-cell regulatory network inference and clustering (SCENIC) analysis revealed that TNF (25) signaling pathway associated regulons such as Nfkb1, Nfkb2 and Interferon alpha and gamma signaling pathway associated regulons such as Irf1, Irf7, and Irf8, were enriched in PTC-S1-new and PTC-injured (**Figure 2D**). We then analyzed the transcription characteristic between PTC-new and normal PTCs, and explored the difference between the PTC-new and normal PTCs (**Figures 3A–D**). Interestingly, most up-regulated genes or transcription factors in PTC-new were kidney injury or repair regulated genes such as Klf4 (25), Klf6 (26), Neat1 (27), Malat1 (28), and Egr1 (29) (**Figures 3A**–**D**). Compared to normal PTCs, the PTC-new exhibited a high level of MAPK, TNF signaling pathway and associated genes Dusp1 and Jun (**Figure 3E**), which also supported the findings that PTC-new were pro-inflammatory.

### Trajectory Analysis of PTCs Subtypes

**Figure 4:**
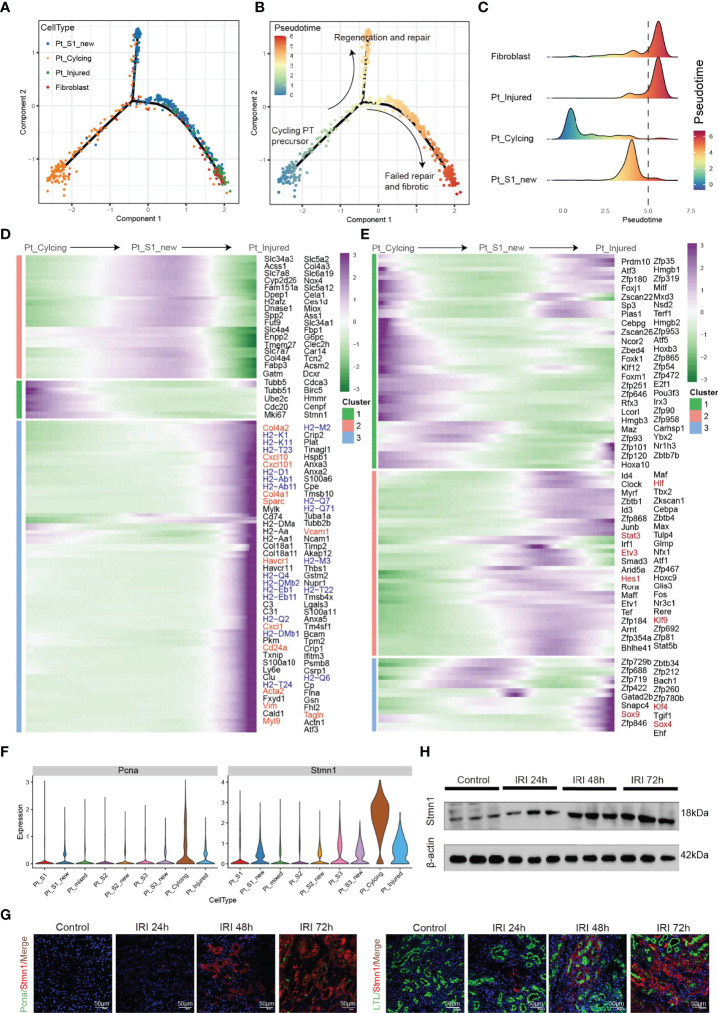
Pseudo-time trajectory analysis among PTC. **(A–C)** Potential differentiation routines among PTC-S1-new, PTC-cycling, PTC-injured, and fibroblast. **(D, E)** Pseudo-temporal expression dynamics of specific representative genes **(D)** and transcriptome factors **(E)** alongside AKI progression. **(F)** The expression of cycling marker Stmn1 and Pcna in PTC subtypes. **(G)** Immunostaining showing the distribution of cycling PTC alongside the AKI progression. **(H)** Western blot showed the expression of Stmn1 with the time-dependent effect of IRI in the mouse kidney.

Tissue repair and regeneration are very complex biological events, where successful attainment requires far more than mere cell division because the proliferative cells may replace the injured cells and promote repair (7, 30). We observed that the portion of PTC-cycling cells elevated along with the AKI (**Figure 1E**) and highlighted the important role of proliferative PTC-cycling cells in AKI repair. We employed the pseudo-time trajectory analysis (31) based on gene expression simulation to infer the cellular differentiation routines or potential transition between PTC-S1-new, PTC-injured, PTC-cycling, and fibroblast (**Figures 4A**–**C**). The pseudo-time trajectory axis indicated that PTC-cycling cells could differentiate into PTC-S1-new and part of PTC-S1-new cells may then turned into PTC-injured and fibroblast (**Figures 4B, C**). Pseudo-temporal expression dynamics of specific representative genes (**Figure 4D** and **Supplementary Figure 5**) and transcriptome factors (**Figure 4E**) also marked the progression of PTC-cycling cells into PTC-S1-new, PTC-injured, and finally acquired fibrotic phenotype. The results presented here delineate the potential translation paths of PTC subtypes and PTC-S1-new may be an important node that determines fibrotic and normal repair. We also revealed injured and repair associated genes and transcription factors such as Hif (32), Irf1 (33), Klf4 (25), Hes1 (34), and Klf6 (26) (**Figures 4D, E**) involved in AKI progression, which may be important biomarkers for screening AKI progression. We also found PTC-cycling and part of PTC-new expressed proliferative markers such as Stmn1 and Pcna (**Figure 4F**). The immunostaining and Western Blot results showed that the expression of cycling markers Stmn1 and Pcna increased along with the AKI progression (**Figures 4G, H**), which validated the important role of cycling PTCs in AKI progression and renal regeneration.

### Interplay Between PTCs Subtypes and Infiltrating Immune Cells

**Figure 5:**
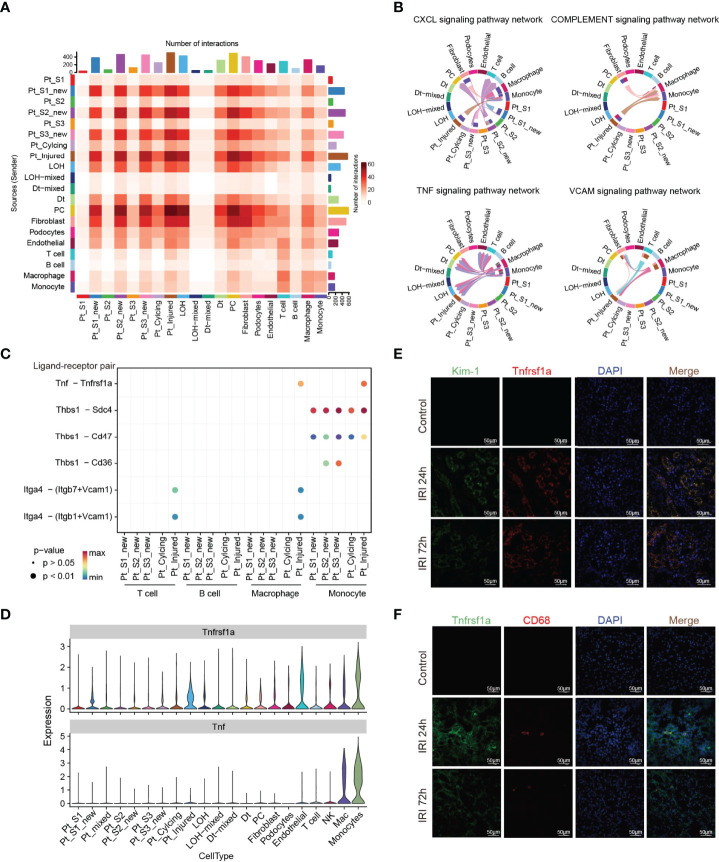
Intercellular crosstalk analysis in AKI microenvironment. **(A)** Number of inferred interactions in 21 cell types. **(B, C)** Enriched immune signaling pathway networks **(B–D)** and significant ligand-receptor Tnf-Tnfrsf1a pair **(C, D)** in injured PTC, PTC-new and immune cells. **(E)** Immunostaining showing the spatial distribution of injured PTC marker Kim-1 and Tnfrsf1a alongside AKI timepoints. **(F)** Immunostaining showing the spatial distribution of CD68 expressing myeloid cells and Tnfrsf1a expressing PTCs alongside AKI timepoints.

Immune response-mediated kidney injury is an important factor in the progression of AKI but the detail molecular mechanism requires further investigation. As described above, our single cell transcriptomic analysis revealed the PTC-injured, part of PTC-new exhibited inflammatory, fibrotic features, and the portion of immune cells elevated along with with AKI progression, indicating the important role of the immune cell in AKI. We therefore employed the CellChat (35) package to infer the cellular interplay mechanism between PTC-new, PTC-injured, and infiltrating immune cells. Interestingly, the interaction node numbers in PTC-S1-new and PTC-injured were the highest among PTCs (**Figure 5A**) and these two subtypes interacted closely with infiltrating immune cells such as T cells, macrophage, and monocytes (**Figure 5A**). Intercellular crosstalk analysis revealed that the inflammatory signaling pathways such as CXCL, TNF, VCAM, and Complement were more enriched between PTC-injured, macrophage, and monocytes (**Figures 5B**–**D** and **Supplementary Figure 6**). Further ligand-receptor analysis revealed that immune signal axis such as Tnf−Tnfrsf1a, Itga4-Vcam1, Thbs1−Sdc4, Cxcl1−Cxcr2 might participate in the intercellular crosstalk between injured PTCs, macrophage, and monocytes (**Figures 5B**–**D** and **Supplementary Figure 6**). Additionally, immunostaining confirmed that the injured PTCs expressed a high level of TNFRSF1A, with co-expression of injured PTC marker Kim-1 (**Figure 5E**). We also found that CD68 positive myeloid cells interplayed closely with Tnfrsf1a positive PTC-injured cells (**Figure 5F**), highlighting the important role of infiltrating immune cell in AKI progression.

### Role of TNFRSF1A-TNF Signaling Axis in Kidney Injury

**Figure 6:**
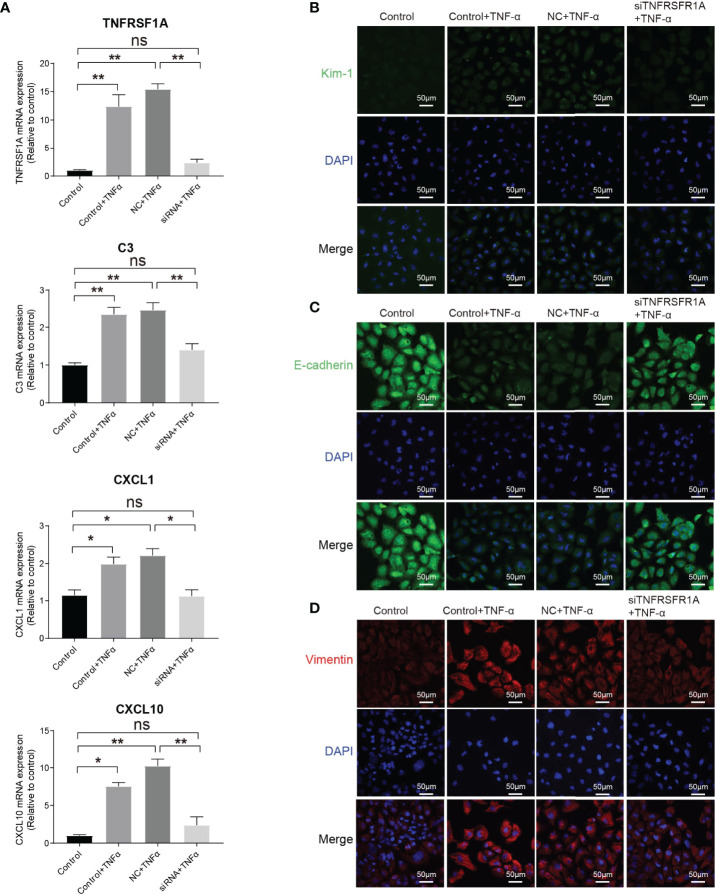
Role of TNF/TNFRSF1A in AKI. **(A)** Expression level of TNFRSF1A and inflammatory signatures C3, CXCL1 and CXCL10 when receiving TNF-α stimulation or not. **(B–D)** Immunostaining showing the expression of injury marker Kim-1 **(B)**, PTC marker (E-cadherin, C) and trans-differentiation marker (Vimentin, D) when receiving TNF-α stimulation or not. Data are performed as means ± SD for three independent experiments. One-way ANOVA with Turkey *post hoc* tests was used for three or more group comparisons. *p<0.05, **p<0.01. ns, Not Statistically Significant.

The intercellular crosstalk analysis and the immunostaining validation experiment provided evidence that the infiltrating macrophage and monocyte interacted closely with PTC-injured through the Tnf−Tnfrsf1a axis (**Figures 5B**–**F**). To investigate the role of the Tnf−Tnfrsf1a axis in kidney injury, we constructed TNFRSF1A silencing (**Supplementary Figure 7**). Quantitative PCR revealed that exogenous TNFα recombinant protein stimulation significantly promoted PTCs TNFRSF1A mRNA expression, while no significant changes were observed in the siTNFRSF1A + TNFα group (**Figure 6A**). We further examined other inflammation-related indicators and found that TNF-α stimulation significantly increased the expression of inflammatory signatures such as C3, CXCL1, and CXCL10 in PTCs, while no significant expression changes in the siTNFRSF1A + TNFα group compared to the control group (**Figure 6A**). We also found that TNF-α stimulation significantly promoted the expression of the PTC injury marker (Kim-1, **Figure 6B**) and the renal epithelium trans-differentiation marker (Vimentin, **Figure 6C**), while it downregulated the expression of renal tubular epithelial cells marker (E-cadherin, **Figure 6D**). These results suggest that the TNF-α dramatically promoted kidney inflammation and injury and specific depletion of TNFRSF1A would abrogate the injury (36–39).

## Discussion

AKI is a potentially fatal disease which can trigger or exacerbate CKD, and is associated with high mortality and morbidity. The progression of AKI to CKD usually undergoes immune cell infiltration, renal cells phenotypic alterations, and then ECM deposition which leads to pathological fibrosis. Immune response-mediated kidney injury is an important factor in the progression of AKI, which determines normal repair or pathological repair when it progresses to CKD. Comprehensively dissecting the key cellular player and intercellular crosstalk target associated with AKI pathobiology is critical for underlying mechanisms to determine the transition from acute to chronic injury, precision diagnosis, and develop novel therapy strategies for AKI.

Several studies have characterized diverse immune cell subtypes and associated features along with the AKI progression, identified renal repair, and injury associated myeloid and T cell subtypes (9, 12, 24). Conway BR et al., utilized scRNA-seq to uncover the myeloid cell diversity and found that monocyte acquired proinflammatory, profibrotic phenotype that expressed Arg1 and the Mmp2^+^ macrophages expanded during repair (9). Fernanda et al., reported that expansion of tissue-resident IL-33R^+^ and IL-2Ra^+^ regulatory T cells (Tregs), before injury, protected the kidney from injury and fibrosis (12). Some studies focused on the diversity of PTC phenotype, the mixed-identity PTCs, injured PTCs, and the identity of failed-repair PTCs (24, 40–42), as well as novel genes and potential pathologic intercellular crosstalk targets such as Ahnak, Sh3bgrl3, Col18a1, Krt20, Vcam1, and Ccl2 (42). These studies have broadened our knowledge of understanding AKI onset and progression. However, the cellular origin of PTC-injured, the molecular characteristics of injured PTCs, the dynamic molecular change along with AKI progression, and how infiltrating immune cells influence the process of AKI and repair, remains poorly investigated.

In this study, we employed unbiased scRNA-seq to systematically resolve the cellular atlas and microenvironment associated with AKI progression. We identified three PTC-new subtypes, and the mixed PTCs indicated that renal epithelial cells may exhibit diverse transcriptome phenotypes in response to injury. Interestingly, the PTC-new cells exhibited impairment of metabolic features and upregulation of proinflammatory and pro-fibrotic features compared to the normal PTCs. Additionally, the kidney injury associated genes, (TFs, pathways, and regulons) were enriched in PTC-new and mixed PTCs, with the highest expression level in the PTC-injured subpopulation. Further cellular differentiation and trajectory analysis indicated that PTC-cycling cells may turn into PTC-S1-new, and then to PTC-injured, and lastly became fibrotic. Importantly, we found that infiltrating immune cells (mainly as monocyte and macrophage) interact closely with PTC-injured and influence the process of AKI progression mainly through inflammatory signaling pathways such as CXCL, complement, VCAM, and TNF. Immunostaining also validated that injured PTCs expressed a high level of TNFRSF1A and injury marker Kim-1, and functional experiments revealed that the exogenous addition of TNF-α dramatically promoted kidney inflammation and injury and specific depletion of TNFRSF1A would abrogate the injury.

Taken together, our study provides a comprehensive cellular atlas for depicting the AKI microenvironment and key molecular pathways that are perturbed in AKI. The results presented here highlighted Tnf−Tnfrsf1a (36–39) and Cxcl1−Cxcr2 pathways (43, 44) and were potential targets of kidney injury, which may be a benefit for AKI repair.

## Materials and Methods

### Ethics Statement

This study was conducted according to the principles expressed in the declaration of Helsinki. Ethical approval was obtained from the Ethics Committee of the Chinese People’s Liberation Army General Hospital with number 2019-X5-65.

### Mice, Surgical Procedures, and Serum Analysis

C57BL/6 mice (20–25 g) were purchased from the Animal Center of Chinese PLA General Hospital. All animal procedures were approved by the Institutional Animal Care and Use Committee at the Chinese PLA General Hospital and Military Medical College. The 24 male mice were randomly assigned to two groups: 18 mice underwent bilateral renal ischemia and reperfusion surgery (the AKI group), and the remaining 6 mice underwent sham surgery (the control group). Renal ischemia (28 min) and reperfusion and renal sham surgery were performed as described previously. At 24h, 48h, and 72h after reperfusion, blood and kidney samples were harvested for further processing. Kidney injury was assessed by measuring the levels of serum creatinine and blood urea nitrogen by a quantitative colorimertic assay and calculating the changes in the levels. Blood samples were collected from the vena cava at the indicated time points and the serum was separated by centrifugation at 3,000 rpm for 15 min at 4°C; and then serum Cr and BUN examinations were performed. Serum Scr were measured with the DuantiChromTM Creatinine Assy Kit (Bioassy system, US, DICT-500) by the improved Jaffe method, while the BUN concentration levels were detected with the DuantiChromTM Urea Assy Kit (Bioassy system, US, DIUR-500) by the improved Jung method. For the measurements of serum creatinine and BUN, six animals were analyzed per group and three technical repeats were analyzed from each example.

### Histopathological Examination

A quarter of the kidney was fixed in 4% formaldehyde, dehydrated, and embedded in paraffin. Tissue sections (4 μm) were stained with periodic acid–Schiff (PAS). Histological examinations were performed in a blinded manner for acute tubular necrosis (ATN) scores regarding the grading of tubularnecrosis, cast formation, tubular dilation, and loss of brush border as described previously. Fifteen non-overlapping fields (400×) were randomly selected and scored as follows: 0, none; 1, 1–10%; 2, 11–25%; 3, 26–45%; 4, 46–75%; and 5,>76%.

### Western Blot

Mouse renal tissue or cells were lysed with RIPA lysis buffer containing protease inhibitors (1 µg/mL leupeptin, 1 µg/mL aprotinin and 100 µmol/L PMSF). After a 30 minute incubation, the samples were centrifuged at 13800 g at 4°C for 30 minutes. The protein concentration was determined by a BCA protein assay kit (Thermo Fisher Scientific, USA). Approximately 40 µg of protein from each sample was separated by 8%- 15% SDS- PAGE. The samples were transferred from the SDS- PAGE gels to membranes. The membranes were blocked and incubated in antibodies against Stmn1 (1:1000, abcam, ab52630), GAPDH (1:10000, proteintech, 60004-1-Ig) and β-actin (1:10000, proteintech, 66009-1-Ig) overnight at 4°C. Finally, the membranes were incubated with secondary antibody at room temperature for 2 hours. ImageJ was used for blot analysis. All experiments were repeated three times.

### Cell Culture

HK-2 cells were purchased from ATCC (CRL-2190). Cells were inoculated into 24-well plates lined with coverslips and cultured in DMEM/F12 medium containing 10% FBS at 37°C in a 5% CO2 incubator. Small interfering RNA (siRNA) was produced by Gene Pharma (Gene Pharma Co.,Ltd., China). The target sequence of siRNA for human TNFRSF1A was as follows: Sense: 5’-GGUGGAAGUCCAAGCUCUATT-3’, Antisense: 5’-UAGAGCUUGGACUUCCACCTT and NC was used as negative control. The cells were divided into 4 groups: Control group, Control+TNFα group (human-derived TNFα recombinant protein treated group), NC+TNFα group (no significant RNA transfection + human derived TNFα recombinant protein treated group), and siRNA+TNFα group (siRNA transfected with TNFRSF1A + human derived TNFα recombinant protein treated group). At approximately 70% cell fusion, the corresponding si-TNFRSF1A and nonsense RNAs were transfected using Lipofectamine_RNAiMAX for the siRNA+TNFα and NC+TNFα groups, and 15 ng/ml of human-derived TNFα recombinant protein (Biolegend, Cat717904) was given 24 h post-transfection to stimulate For the Control+TNFα group, only 15ng/ml of human TNFα recombinant protein was administered for 24 h. The Control group was left untreated.

### qRT-PCR Analysis

Total RNA was extracted from cells using TRIzol and reversely transcribed to cDNA using ProtoScript^®^ II First Strand cDNA Synthesis Kit (E6560S, NEB) according to the manufacturer’s instructions. qRT-PCR was conducted using PowerUp SYBR Green Master Mix (Applied Biosystems, Foster City, CA, USA) and performed using the CFX-96 (Bio-Rad, USA). The cycling parameters were as follows: 10 min at 95°C, 45 cycles of 10 s at 95°C, and 30 cycles at 58°C following the manufacturer’s instructions. Data were performed as fold induction relative to the control group and the relative mRNA level of target gene was analyzed by the formula 2–ΔCt (ΔCt = Ct ^target^ - Ct ^18S^). The primer sequences are shown in **Supplementary Table S1**.

### Immunofluorescence Staining

For mouse samples, tissue specimens were fixed in 4% paraformaldehyde and embedded in paraffin. Tissue samples were then cut at 4 μm thickness and sequentially treated with 1% SDS and normal goat serum before being incubated with Anit-PCNA (abcam, ab29, 1:100), Anti-Stathmin 1 (abcam, ab52630, 1:100), Anti-Kim-1 (R&D, AF1817, 1:400), Anti-CD68(abcam, ab125212, 1:200), and TNFR1(proteintech, 60192-1-Ig, 1:100) overnight at 4°C. The sections were washed and probed with Cy3-conjugated secondary antibody (red) and FITC- conjugated secondary antibody (green) or LTL (Vector Laboratories, FL-1321, 1:400) at room temperature for 1 hour. DAPI was added to stain the nuclei. The tissue sections were imaged by confocal fluorescence microscopy. Each experiment was repeated three times. For cell samples, cells were washed with PBS and fixed with 4% paraformaldehyde for 15 min. The cells were then permeabilized with 0.2% Triton X-100 for 15min and blocked with 5% BSA in PBS for 1h at room temperature. Cells were then incubated overnight at 4° with Anti-E-Cadherin (R&D, AF748, 1:400), Anti-Kim-1 (R&D, AF1817, 1:400), and Anti-Vimentin (abcam, ab92547, 1:200). After cells were washed with PBS three times and incubated with secondary antibodies (Cy3 conjugated anti-rabbit antibody and FITC conjugated anti-Goat antibody) for 1h at room temperature, they were washed again and mounted onto glass slides with Flouroshield Mounting Medium with DAPI (abcam, ab104139). The cells were imaged by confocal fluorescence microscopy.

### Sample Processing and Cell Sorting

Surgical resected fresh kidney samples were minced and enzymatically digested to obtain single-cell suspensions. Briefly, the samples were minced into <1 mm3 pieces and digested with 5 mL digestion buffer containing DNase I (1 mg/mL, Sigma) and collagenase IV (2 mg/mL, Sigma) for 30 min at 37°C. Next, the resulting suspension was mixed with 5 mL 2% FBS/PBS, filtered with a 70-µm pore size cell strainer (Corning, USA), and centrifuged at 300 g for 5 min at 4°C. After removal of the supernatant, the cell pellet was resuspended in 2 mL red blood cell lysis buffer (BD) for 3 min at room temperature and centrifuged at 300 g for 5 min at 4°C. Then, the supernatant was removed, the cell pellet was resuspended in 100 μL 1% BSA/PBS, and the cells were incubated with 7-AAD (BioLegend) before cell sorting. For the following single-cell library preparation and sequencing, we sorted and collected 7-AAD negative live cells using a BD FACSAria II (BD).

### Single-Cell mRNA Library Preparation and Sequencing

The single-cell suspensions were prepared from pools of six animals from each group and then loaded onto a microfluidic chip to generate the complementary deoxyribonucleic acid (cDNA) library using a commercial 10x Genomics platform (10x Genomics, Pleasanton, CA, USA). Single-cell transcriptome amplification and library preparation were performed using the Single-Cell 3’ Library Kit v3 (10x Genomics) by Capitalbio Technology Corporation according to manufacturer’s instructions. Then, the libraries were pooled and sequenced across six lanes on an Illumina NovaSeq 6000 system (Illumina, Inc., San Diego, CA, USA).

### Pre-Processing of scRNA-Seq Data

The raw sequencing FASTQ files were aligned to the mm10 reference genome using the cellranger count function of CellRanger (10X Genomics, v5) to produce a gene expression matrix *via* the STAR algorithm. Then, the raw gene expression matrices were processed by the Seurat (45) R package (version 4.0.0). As the kidney is a highly metabonomic organ, the renal cells serve important roles in energy metabolism, and these cells featured a high ratio of mitochondrial genome transcripts (22). Therefore, low-quality cells were removed according to the following criteria: cells that had fewer than 2,001 unique molecular identifiers (UMIs), more than 6,000 or less than 501 expressed genes, or over 50% of UMIs derived from the mitochondrial genome as described previously. Included genes were expressed in at least ten cells in a sample. We removed potential cell doublets using the DoubletFinder (46) R package. The single cell transcriptome expression matrices of the remaining high-quality cells were integrated with the “RunFastMNN” function of SeuratWrappers package, normalized to the total cellular UMI count, and scaled (scale.factor = 1e4) by regressing out the total cellular UMI counts and percentage of mitochondrial genes. Then, we selected highly variable genes (HVGs) for principal component analysis (PCA), and the top 30 significant principal components (PCs) were selected for Uniform Manifold Approximation and Projection (UMAP) dimension reduction and visualization of gene expression.

### Determination of Cell Type

We calculated the differentially expressed genes (DEGs) of each cell subcluster by the “FindAllMarker” function with default parameters provided by Seurat. The cell types and subtypes were annotated according to their expression of the known canonical marker genes of the respective cell types. Cell subclusters with similar gene expression patterns were annotated as the same cell type.

### Trajectory Analysis

To illustrate the potential cellular differentiation routines (31) and dissect the origin of PTC-injured subpopulation, the PTC-injured, PTC-cycling, PTC-S1-new were selected and the top 150 signature genes were calculated by differentialGeneTest function provided by Monocle algorithm. The cell differentiation trajectory was inferred with the default parameters of Monocle after dimension reduction and cell ordering. Then, the ‘DDRTree’ function was used for dimensionality reduction, and the ‘plot_cell_trajectory’ function was used for visualization.

### Single-Cell Regulatory Network Analysis

To explore the single-cell gene regulatory network among different PTC cell subclusters, we analyzed the differentially expressed transcriptome factors with a standard pipeline implemented in R using the SCENIC (47) R package (https://github.com/aertslab/SCENIC). Briefly, the gene expression matrices of the cell subclusters were estimated using GENIE3 to build the initial co-expression gene regulatory networks (GRN). Then, regulon data were analyzed using the RcisTarget package to create TF motifs. The regulon activity scores of each cell were calculated from the AUC by the AUCell package. We filtered the regulons with a correlation coefficient >0.3 with at least one other regulon and the regulons that were activated in at least 30% of the cell subclusters were selected for the subsequent visualization.

### Pathway Analysis

DEGs with |logFC| > 0.5 and adj.p.val < 0.05 were adopted for GO enrichment analysis. The compareCluster function in the clusterProfiler R package was used to find different enriched GO terms between distinct PTC subclusters. To assess the different pathways between distinct PTC subsets, GSVA (48) analysis was performed using the hallmark gene sets provided by the Molecular Signatures Database (MSigDB) and calculated with a linear model offered by the limma package.

### Intercellular Crosstalk Analysis

To explore potential intercellular crosstalk between infiltrating immune cells and PTC-injured subpopulation, we implied the ligand-receptor distribution and expression of infiltrating immune cells and PTC-injured subpopulation with a standard pipeline implemented in R using CellChat (35) R package, as previously reported. We chose the receptors and ligands expressed in more than 10% of the cells in the specific cluster for subsequent analysis. The interaction pairs whose ligands belonged to the VCAM, TNF, Complement, and CXCL families were selected for the evaluation of intercellular crosstalk between the distinct PTC subpopulations and infiltrating immune cells.

### Data Availability

The accession number for the raw data reported in this paper have been deposited in the Genome Sequence Archive (GSA) under accession number CRA006298 and the processed data can be accessed in https://ngdc.cncb.ac.cn/omix/view/OMIX001004.

### Statistical Analysis

Data were analyzed using the non-parametric Mann-Whitney U test of two group and one-way ANOVA with Tukey’s multiple comparisons test for three or more group. *P <*0.05 was statistically significant. Statistical analyses were performed using GraphPad Prism 5.0 and data are expressed as mean ± SD.

## Data Availability Statement

The datasets presented in this study can be found in online repositories. The names of the repository/repositories and accession number(s) can be found below: GSA, https://ngdc.cncb.ac.cn/gsa/browse/CRA006298; OMIX, https://ngdc.cncb.ac.cn/omix/release/OMIX001004.

## Ethics Statement

The animal study was reviewed and approved by the Ethics Committee of the Chinese People’s Liberation Army General Hospital with number 2019-X5-65.

## Author Contributions

MZ was responsible for the single cell analysis, and the writing of the manuscript. LLW collected the samples and performed functional experiment with the help of YD, TW, YZ, JL, and PC. LQW, JW, and XC were responsible for the study concept, design, and interpretation. All authors participated in the discussion. All authors contributed to the article and approved the submitted version.

## Funding

This work was supported by National Key R&D Program of China (2017YFA0103203, 2017YFA0103200), National Natural Science Foundation of China (82030025), Military Medical Key Projects (BLB19J009), and the National Key R&D Program of China (2018YFA0108803).

## Conflict of Interest

The authors declare that the research was conducted in the absence of any commercial or financial relationships that could be construed as a potential conflict of interest.

## Publisher’s Note

All claims expressed in this article are solely those of the authors and do not necessarily represent those of their affiliated organizations, or those of the publisher, the editors and the reviewers. Any product that may be evaluated in this article, or claim that may be made by its manufacturer, is not guaranteed or endorsed by the publisher.
