# Supplementary material for: Single Cell Dissection of Epithelial-Immune Cellular Interplay in Acute Kidney Injury Microenvironment
Source: Front Immunol. 2022 May 4;13:857025. doi: 10.3389/fimmu.2022.857025 (PMC9114878; doi:10.3389/fimmu.2022.857025)
Supplement: Supplementary file 1 [file DataSheet_1.pdf]

Supplementary figure legends

Supplementary figure 1: Evaluation of renal injury on 24h, 48h, 72h after IRI by serum analysis. (A) Serum Creatine concentrations. (B) Serum BUN concentrations. Data are performed as means ± SD, (n=6). One-way ANOVA with Turkey post hoc tests was used for three or more group comparisons. *p<0.05, **p<0.01.

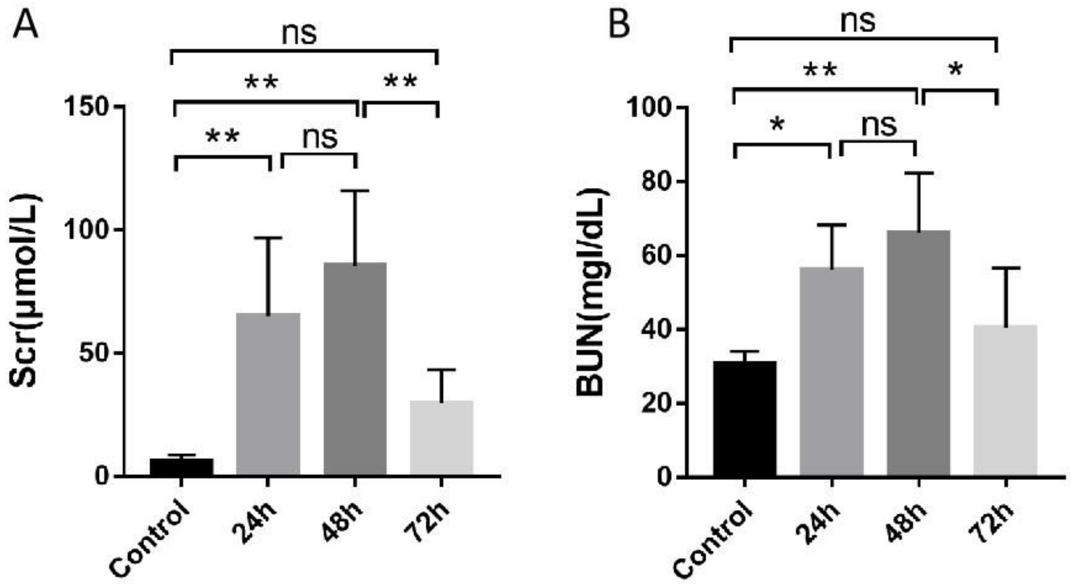

Supplementary figure 2 Violin plots showing gene counts and UMI counts of 21 cell types.

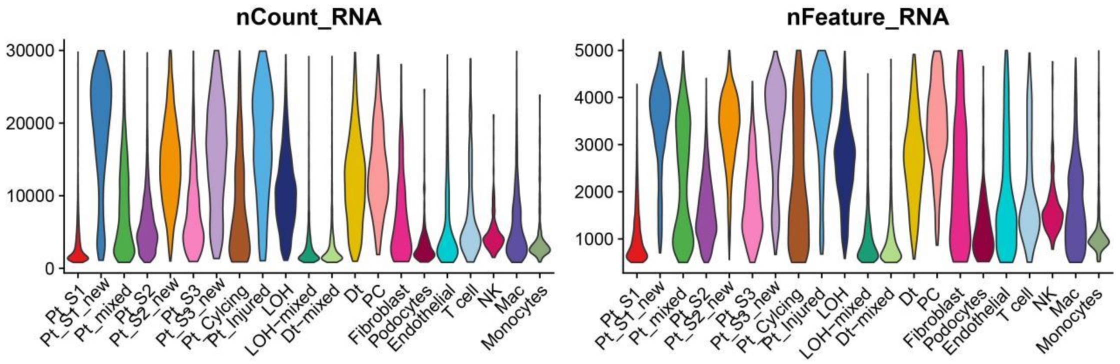

Supplementary figure 3 Violin plots showing antigen processing and presentative genes among 21 cell types.

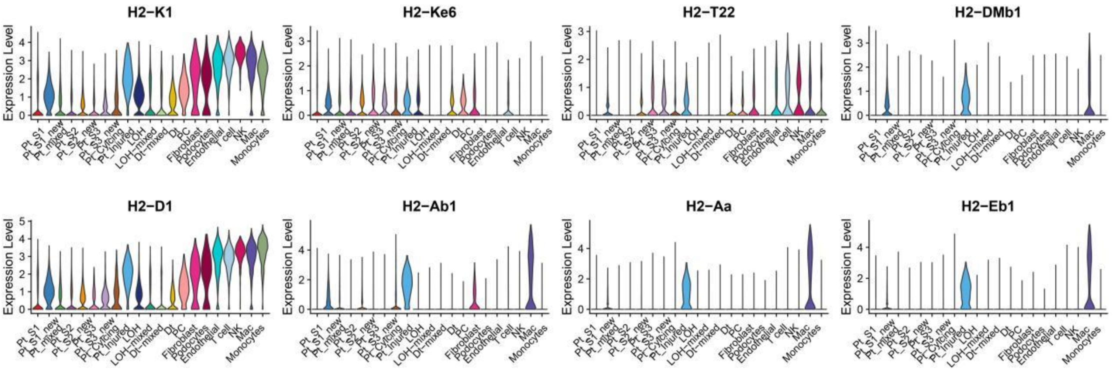

Supplementary figure 4 Enriched signaling pathways in PTC-new, injured PTC and normal PTC.

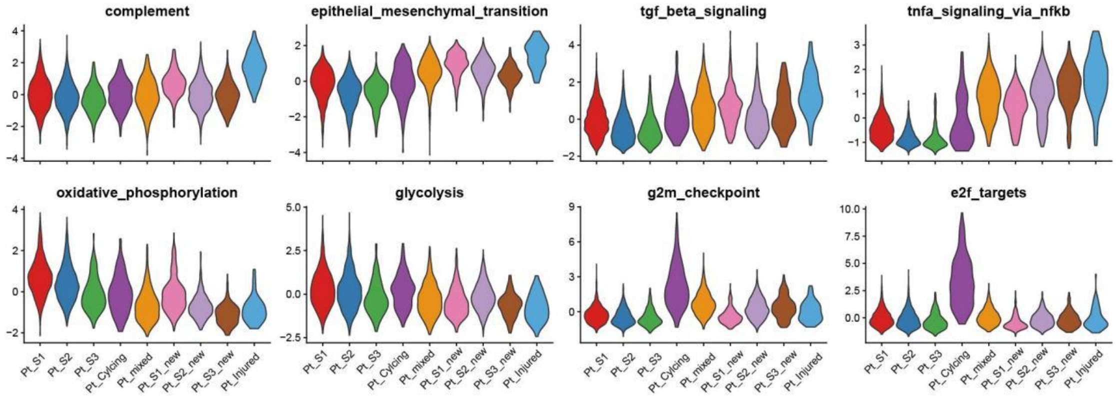

Supplementary figure 5 Pseudo-temporal expression dynamics of specific representative genes Stmn1, Havcr1, Cxcl1,Cxcl10, Col4a1, and Acta2 alongside AKI progression

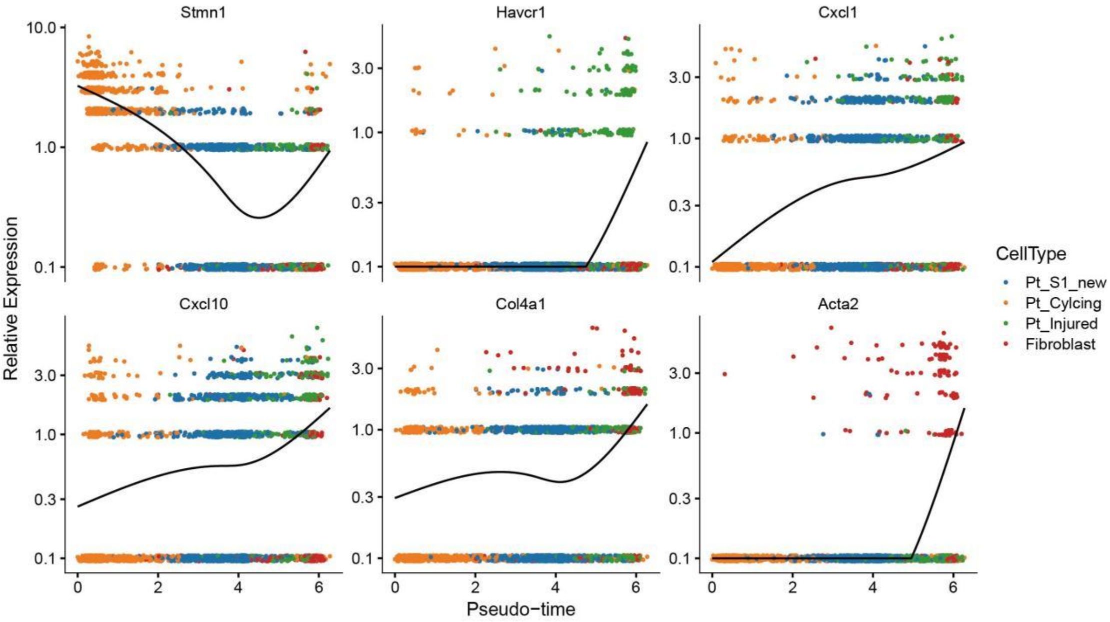

Supplementary figure 6 The expression level of CXCL signaling pathway network (A) and ligand-receptor pair (B) in injured-PTC, PTC-new, and immune cells.

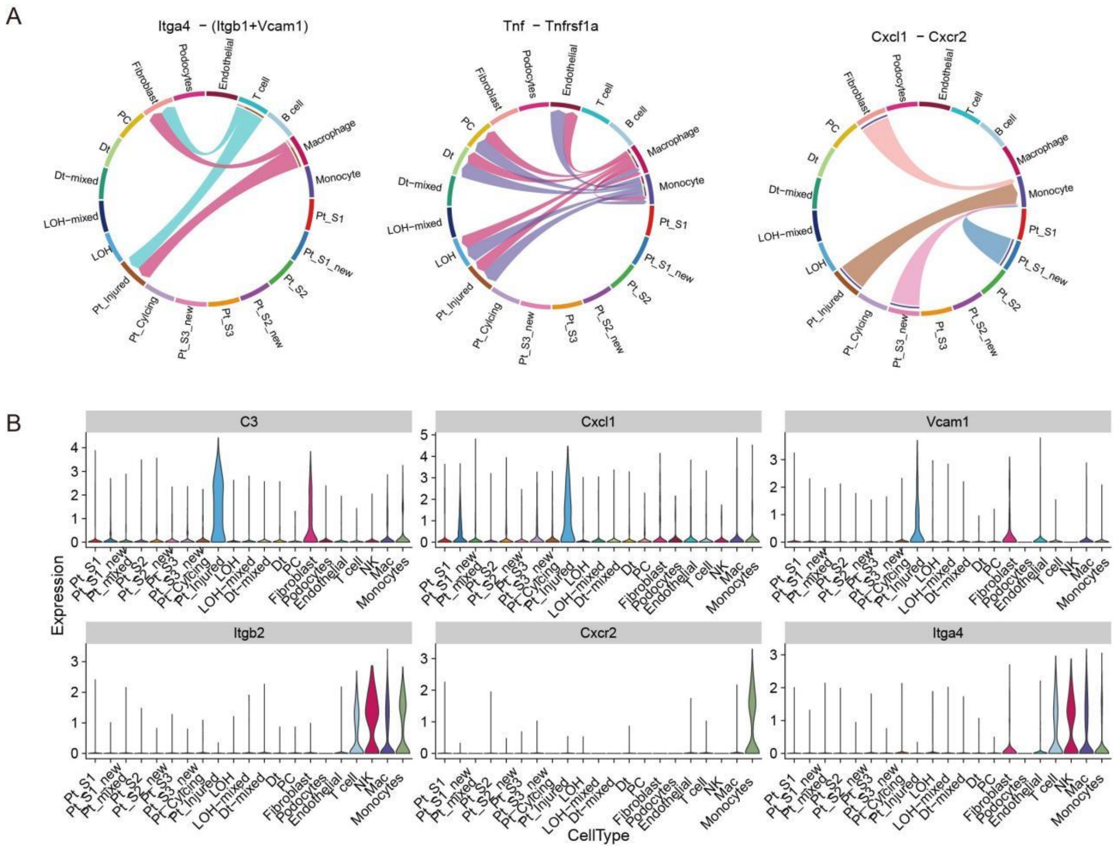

Supplementary figure 7： The expression of TNFRSF1A protein following the siRNA.

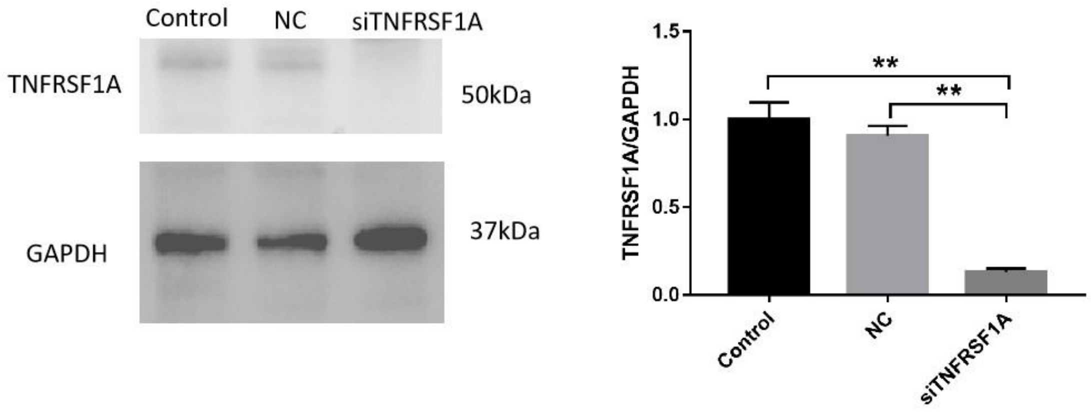

Supplementary table 1 Primer sequences for qPCR analysis.

Gene	Species	Nucleotide sequence
TNFRSF1A	Human	5’- tgctgtaccaagtgccacaa-3’ (F)
		5’- tgacccatttcctttcggca-3’ (R)
C3	Human	5’- ctgcccagtttcgaggtcat-3‘ (F)

		5'- caatcggaatgcgcttgagg-3' (R)
CXCL1	Human	5'- caaagtgtgaacgtgaagtccc-3' (F)
		5'- gttggatttgtcactgttcagc-3' (R)
CXCL10	Human	5'- tgccattctgatttgctgcc-3' (F)
		5'- tggacaaaattggcttgcagg-3' (R)
18S	Human	5'- gtaacccgttgaaccccatt-3' (F)
		5'- ccatccaacggtagtagcg-3' (R)
